# Cotton Fabric Coated with Conducting Polymers and its Application in Monitoring of Carnivorous Plant Response

**DOI:** 10.3390/s16040498

**Published:** 2016-04-08

**Authors:** Václav Bajgar, Marek Penhaker, Lenka Martinková, Andrej Pavlovič, Patrycja Bober, Miroslava Trchová, Jaroslav Stejskal

**Affiliations:** 1 Department of Cybernetics and Biomedical Engineering, Faculty of Electrical Engineering and Computer Science, VSB-Technical University of Ostrava, 708 33 Ostrava, Czech Republic; vaclav.bajgar.st@vsb.cz (V.B.); marek.penhaker@vsb.cz (M.P.); 2 Inotex Ltd, 544 01 Dvur Kralove nad Labem, Czech Republic; martinkova@inotex.cz; 3 Department of Biophysics, Centre of the Region Haná for Biotechnological and Agricultural Research, Faculty of Science, Palacky University in Olomouc, 783 71 Olomouc, Czech Republic; andrej.pavlovic@upol.cz; 4 Institute of Macromolecular Chemistry, Academy of Sciences of the Czech Republic, 162 06 Prague 6, Czech Republic; trchova@imc.cas.cz (M.T.); stejskal@imc.cas.cz (J.S.)

**Keywords:** conducting polymers, plant neurobiology, polyaniline, polypyrrole, Venus flytrap

## Abstract

The paper describes the electrical plant response to mechanical stimulation monitored with the help of conducting polymers deposited on cotton fabric. Cotton fabric was coated with conducting polymers, polyaniline or polypyrrole, *in situ* during the oxidation of respective monomers in aqueous medium. Thus, modified fabrics were again coated with polypyrrole or polyaniline, respectively, in order to investigate any synergetic effect between both polymers with respect to conductivity and its stability during repeated dry cleaning. The coating was confirmed by infrared spectroscopy. The resulting fabrics have been used as electrodes to collect the electrical response to the stimulation of a Venus flytrap plant. This is a paradigm of the use of conducting polymers in monitoring of plant neurobiology.

## 1. Introduction

**Figure 1:**
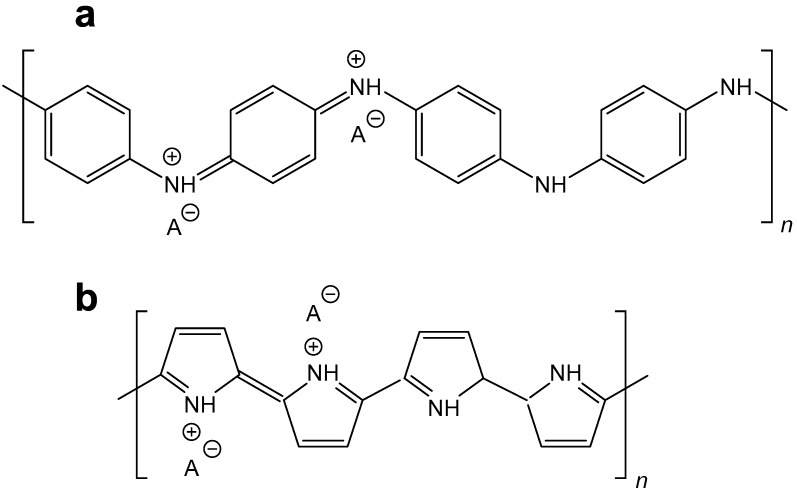
Conducting (**a**) polyaniline and (**b**) polypyrrole salts. A^−^ is an arbitrary anion.

Conducting polymers, such as polyaniline (PANI) and polypyrrole (PPy) (Figure 1), have recently been studied due to the variety of nanostructures they produce [1,2,3] and their application potential in energy storage and energy conversion devices, namely batteries, fuel cells, and supercapacitors. They have recently been applied as adsorbents, conducting inks, heterogeneous catalysts, in corrosion protection, sensors, electromagnetic interference shielding, and many other directions [4,5,6]. The use of conducting polymers in life sciences for monitoring or stimulation of biological objects ranks among the most modern trends [7,8,9].

The attractiveness of conducting polymers has been associated with the ease of their preparation [10]. Conducting polymers, however, are intractable as a rule and their mechanical properties are poor. The deposition of conducting polymers on various supports offers a solution to this problem. Various textiles have recently become coated with conducting polymers [11,12,13,14,15,16,17,18,19]. This is done *in situ*, by immersion of the template fabrics in the aqueous reaction mixture used for the preparation of these polymers by the oxidation of respective monomers.

The use of conducting textiles in wearable electronics, in contact with biological objects, is the obvious target [20]. The cleaning of such fabrics is usually required for such applications. The conducting polymers, however, lose most of its conductivity at physiological conditions or during washing [18,21,22,23], when conducting polymer salts convert to non-conducting bases [10]. The level of conductivity after water laundering was maintained only after the incorporation of graphene oxide [24,25]. It has recently been reported that the problem can be overcome by using dry cleaning instead [18,25].

While the most common conducting polymers, PANI and PPy, have been investigated in numerous papers, the reports on composites comprising simultaneously both polymers are limited [26,27,28,29,30,31,32,33]. Cotton has commonly been used as a substrate for the deposition of conducting polymers [13,17,18,24,34,35,36,37,38,39]. In the present paper, we have studied cotton fabric coated with PANI and subsequently with PPy, or *vice versa*, to see if there is any synergism or improvement in the properties of the resulting system with respect to conductivity and repeated dry cleaning.

The application of the modified textiles in the monitoring of electrical response from the plant stimulation was subsequently tested. Plants usually respond to different environmental stimuli by generation of electrical signals in the form of action and variation potentials [40]. Action potentials in plants are generated in response to cold or touch. They usually have an all-or-nothing character; that is, after a stimulus reaches a certain threshold (which leads to membrane depolarization), further increases in stimulus strength do not change its amplitude and shape. Variation potentials are usually generated in response to damaging stimuli (e.g., cutting or burning). The main difference to action potentials lies in longer, delayed repolarizations and a large range of variation. This signal varies with the intensity of the stimulus and appears to be a local change to a hydraulic pressure [40]. The best known example of electrical signaling in plants is described in the carnivorous plant Venus flytrap (*Dionaea muscipula*) and sensitive plants (*Mimosa pudica*) [41,42]. For testing of the conductive polymers to transduce electrical signals in plants, we chose Venus flytrap because we had previously worked successfully with this species [43].

## 2. Experimental

### 2.1. Cotton Coating with Conducting Polymers

Bleached, plain weave 100% cotton fabric CARLTON (Mileta a.s., Hořice, Czech Republic), specific mass 120 g·m^−2^, sett: 51.2 n·cm^−1^ (warp), 28.0 n·cm^−1^ (weft), yarn count 7.4/2 tex (warp), and 14.5 tex (weft) was used as received. The fabric was coated with PANI by immersion in the reaction mixture containing 0.2 M aniline hydrochloride and 0.25 M ammonium peroxydisulfate [10] or in 0.2 M pyrrole and 0.5 M iron(III) chloride mixture for PPy coating [44]. The coated fabrics were rinsed with 0.2 M hydrochloric acid to remove the adhering polymer precipitate, and dried in air. The polymer powder produced outside the fabrics was collected on filter, washed with acetone, and dried.

Polyaniline-coated or polypyrrole-coated fabrics have been used as substrates for the second coating with PPy or PANI, respectively, and processed as above. Four samples have subsequently been investigated, the cotton being coated with: (1) polyaniline (C+PANI); (2) polypyrrole (C+PPy); (3) polyaniline and polypyrrole (C+PANI+PPy); and (4) polypyrrole and polyaniline (C+PPy+PANI).

In a separate experiment, the PANI powder, instead of PANI-coated cotton, was suspended in the reaction mixture used for the preparation of PPy. The PANI particles were thus coated with PPy, and the resulting composite contained about 50 wt% of each polymer. The coating of PPy with PANI was carried out in a similar manner.

### 2.2. Characterization

The surface morphology of cotton fabric before and after coating with conducting polymers was characterized with the scanning electron microscopy (SEM) using a JEOL 6400 microscope.

Point-to-point surface resistance was measured at 20 °C and 64% relative humidity according to IEC 61340-4-10 with a METRISO^®^2000-ESD Test Instrument (Wolfgang Warmbier, High-Resistance Tester, Toledo, OH, USA) using two-point probe with a replaceable head 844 and conducting rubber tips for rigid materials testing.

The room temperature conductivity of polymer powders was determined by a four-point method in van der Pauw arrangement using a Keithley 220 Programmable Current Source (Keithley Instruments, Cleveland, OH, USA), a Multimeter as a voltmeter and a scanner equipped with a matrix card. The composite powders were compressed at 70 kN with a manual hydraulic press to the pellets 13 mm in diameter and ≈1 mm thick.

Chemical cleaning was conducted according to the standard EN ISO 105-D01 “Textiles: Tests for colour fastness, Part D01: Colour fastness to dry cleaning using perchloroethylene solvent”. Tested samples of 4 × 10 cm^2^ size set in a closed 10 × 10 cm^2^ cotton bag were placed into the closed rotating stainless vessel of 550 mL volume containing 200 mL of perchloroethylene thermostatted at 30 °C and operated at rotation speed 40 rpm for 30 min. The fabrics were then dried in air at room temperature.

### 2.3. FTIR and Raman Spectra

FTIR spectra have been obtained by ATR spectroscopic technique using Golden Gate™ Heated Diamond ATR Top-Plate (MKII Golden Gate single reflection ATR system) (Specac Ltd, Orpington, UK) with Thermo Nicolet NEXUS 870 FTIR Spectrometer (Thermo Scientific, Madison, WI, USA) in a moisture-purged environment equipped with DTGS detector in the wavelength range 400–4000 cm^−1^. Typical parameters used were 256 of sample scans, resolution 4 cm^−1^, Happ-Genzel apodization, potassium bromide beamsplitter. The spectra were corrected for the presence of moisture and carbon dioxide in the optical path.

Raman spectra excited with HeNe 633 nm laser have been collected on a Renishaw inVia Reflex Raman microspectrometer. A research-grade Leica DM LM microscope with an objective magnification ×50 was used to focus the laser beam on the sample placed on an X–Y motorized sample stage. The scattered light was analyzed by the spectroscope with holographic gratings 1800 lines·mm^−1^. A Peltier-cooled CCD detector (576 × 384 pixels) registered the dispersed light.

### 2.4. Plant Material and Culture Condition

Venus flytrap (*Dionaea muscipula* Ellis) plants were cultivated in well-drained peat moss in plastic pots 7 cm in diameter placed in container filled with distilled water to a depth 1–3 cm. Daily temperature fluctuated between 20–35 °C, relative air humidity 50%–70%, and maximum daily irradiance reached max. 1500 μmol·m^−1^·s^−1^ PAR (photosynthetically active radiation).

### 2.5. Monitoring the Electrical Response from Plant

The electrical signals were recorded by a non-invasive device inside a Faraday cage [45,46]. The trap of Venus flytrap was gently enclosed into the clip coated with cotton fabric coated with conducting polymers. The strip of cotton fabric protruded from the clip and was connected to non-polarizable Ag-AgCl surface electrodes (Scanlab systems, Prague, Czech Republic). The reference electrode was submerged in 1–2 cm of water in dish beneath the pot. The electrodes were connected to an amplifier made in-house (gain 1–1000, noise 2–3 mV, bandwidth −3 dB at 10^5^ Hz, response time 10 μs, input impedance 10^12^ Ω). The signals from the amplifier were transferred to an analog-digital PC data converter (eight analog inputs, 12-bit-converter, ±10 V, PCA-7228AL, supplied by TEDIA, Pilsen, Czech Republic), collected every 6 ms. The sensitivity of the device was 13 μV. The mechanical stimulation of trigger hairs in enclosed trap of Venus flytrap was performed by plastic stick and the electrical response in the form of action potential was recorded.

## 3. Results and Discussion

### 3.1. The Coating of Cotton with Conducting Polymers

**Figure 2:**
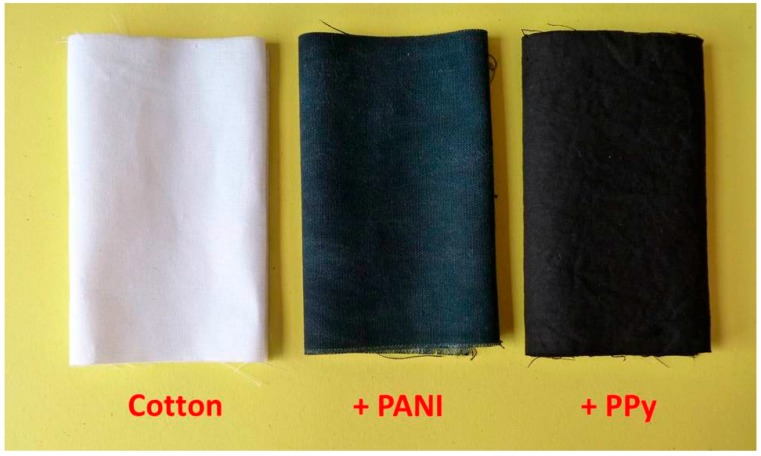
Cotton fabrics before and after the coating with polyaniline or polypyrrole (from left to right).

The *in situ* coating of virtually any surfaces, including textiles, with conducting polymers is based on the immersion of the substrate in the reaction mixture used for their preparation. This is typically the aqueous mixture containing aniline hydrochloride and ammonium peroxydisulfate for PANI [47] or pyrrole and iron(III) chloride for PPy [18,44]. The naked eye immediately observes the difference in the color of textiles (Figure 2); originally white cotton becomes dark green after coating with polyaniline or black after the deposition of PPy.

**Figure 3:**
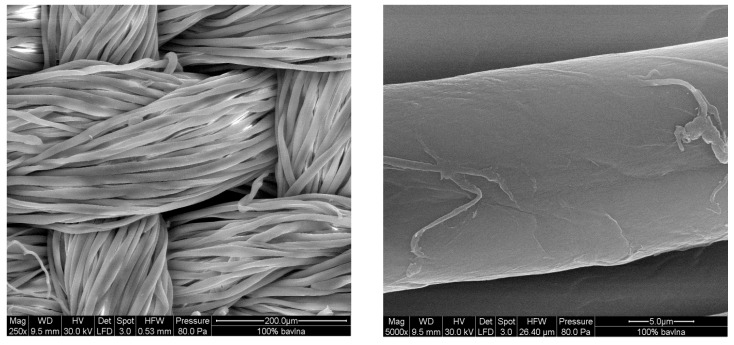
Scanning electron micrographs of cotton fabric (**left**) and its individual fiber (**right**).

**Figure 4:**
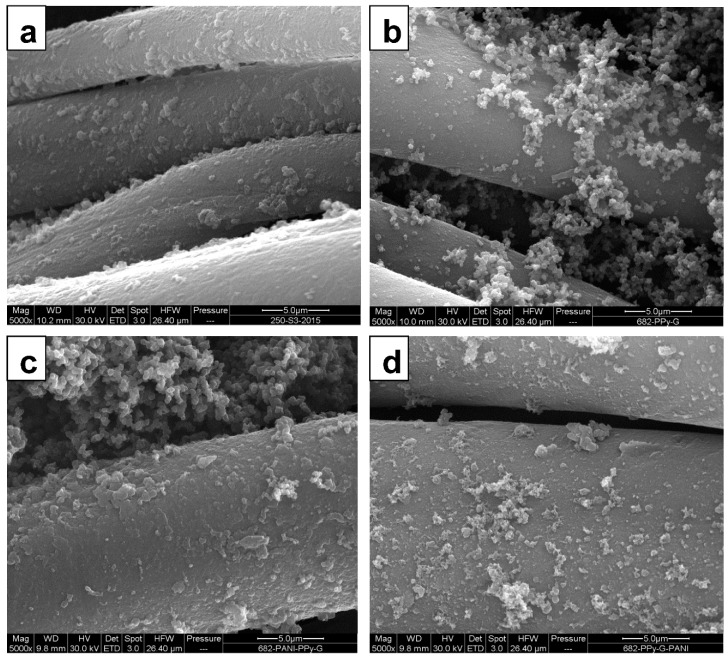
Scanning electron micrographs of cotton fibers coated with: (**a**) PANI; (**b**) PPy; (**c**) PANI+PPy; and (**d**) PPy+PANI.

By using this deposition technique, the cotton fabrics (Figure 3) were coated with PANI (Figure 4a) or PPy (Figure 4b) at first, and subsequently the second polymer was again deposited on top (Figure 4c,d). Scanning electron microscopy reveals the uniform coating of the individual fibers. From the analogy with other substrates, the thickness of such coating is estimated to be close to 100–200 nm [47,48]. Some adhering polymer precipitate is observed on the top of fibers, and especially PPy globules accompany the corresponding coating.

### 3.2. FTIR and Raman Spectra

**Figure 5:**
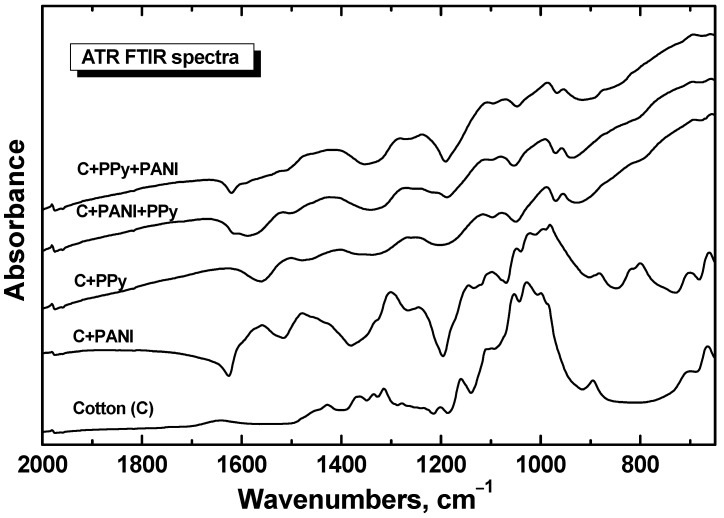
ATR FTIR spectra of cotton fabrics coated with polyaniline (C+PANI) or polypyrrole (C+PPy). Polyaniline-coated cotton was again coated with polypyrrole (C+PANI+PPy) or *vice versa* (C+PPy+PANI). The spectrum of uncoated textile cotton is included.

The microscopy alone need not be convincing enough to demonstrate the uniformity of coating, and further evidence is provided by spectroscopic techniques. The ATR FTIR spectrum of cotton fabric coated with PANI and subsequently with PPy (spectrum C+PANI+PPy in Figure 5) exhibits the main peaks of PPy [44,49,50] (*cf.* the spectrum C+PPy in Figure 5). This means that the coating with PPy is thicker (estimated to be several hundreds of nanometers) than the effective penetration depth of infrared radiation used. The underlying PANI coating is thus not detected in the spectrum.

The spectrum of cotton fabric coated with PPy and then with PANI (spectrum C+PPy+PANI) exhibits, in addition to the main peaks of PPy, also weak features of the spectrum of PANI (*cf.* the spectrum C+PANI in Figure 5) [51]. This corresponds to the fact that the PANI coating is much thinner (*ca.* 100 nm [48]), than the PPy coating and it is smaller than the effective penetration depth of used radiation. Thus, we also observe the bands of the bottom PPy layer in the spectrum. The spectral features of neat cotton are not present in the samples because of strong absorption afforded by conducting polymers.

**Figure 6:**
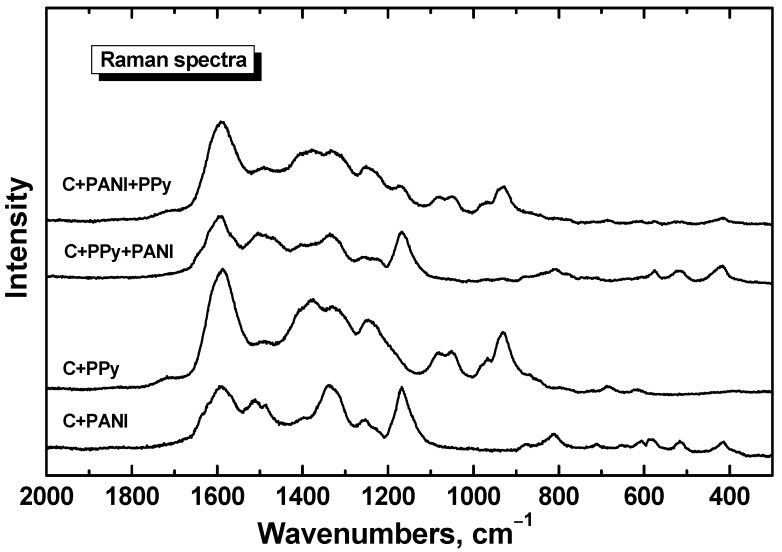
Raman spectra of cotton fabrics coated with polyaniline (C+PANI) or polypyrrole (C+PPy). Polyaniline-coated cotton was again coated with polypyrrole (C+PANI+PPy) or *vice versa* (C+PPy+PANI). Laser excitation wavelength 633 nm.

The situation is similar in the case of Raman spectra. The penetration depth for 633 nm excitation laser line is much lower than the thickness of the both polymer coatings. As a consequence, we observe in the spectrum of cotton fabric coated with PANI and then with PPy (the spectrum C+PANI+PPy in Figure 6) the main peaks of PPy [49,50] (*cf.* the spectrum PPy in Figure 6). In analogy, we observe the main bands of PANI (the spectrum PANI in Figure 6) [51] in the spectrum of cotton fabrics coated with PPy and later with PANI (spectrum C+PPy+PANI in Figure 6). The spectrum of neat cotton displays a strong fluorescence with 633 nm excitation laser line, which is suppressed by polymer coating.

### 3.3. Electrical Properties

Sheet resistivity of PPy coating is considerably lower compared with PANI (Table 1). This is due to thicker coating of PPy, because the conductivity of both polymers measured on compressed pellets is of the same order of magnitude (Table 1). The same reasoning applies to the subsequent second coating where additional resistivity decrease is clearly associated with larger amount of conducting polymers deposited on cotton, rather than with more subtle details, such as the potential interaction between the two polymers. Repeated dry cleaning has led to the 2.0–12.1-fold decrease in sheet resistivity but good level of conductivity was maintained in all cases.

Polyaniline and polypyrrole are regarded as mixed conductors displaying electronic and ionic contribution to the overall conduction [52,53,54]. Such polymers might be potentially suitable to operate as electrical transducers between ionic conductors, such as biological objects, and electronic conductors, such as metals. For that reason, they have been tested below in monitoring the electrical signals afforded by the plant stimulation. It should be noted that water is likely to play an important role in these processes, as it supports the ionic conduction.

### 3.4. Response to Plant Stimulation

**Figure 7:**
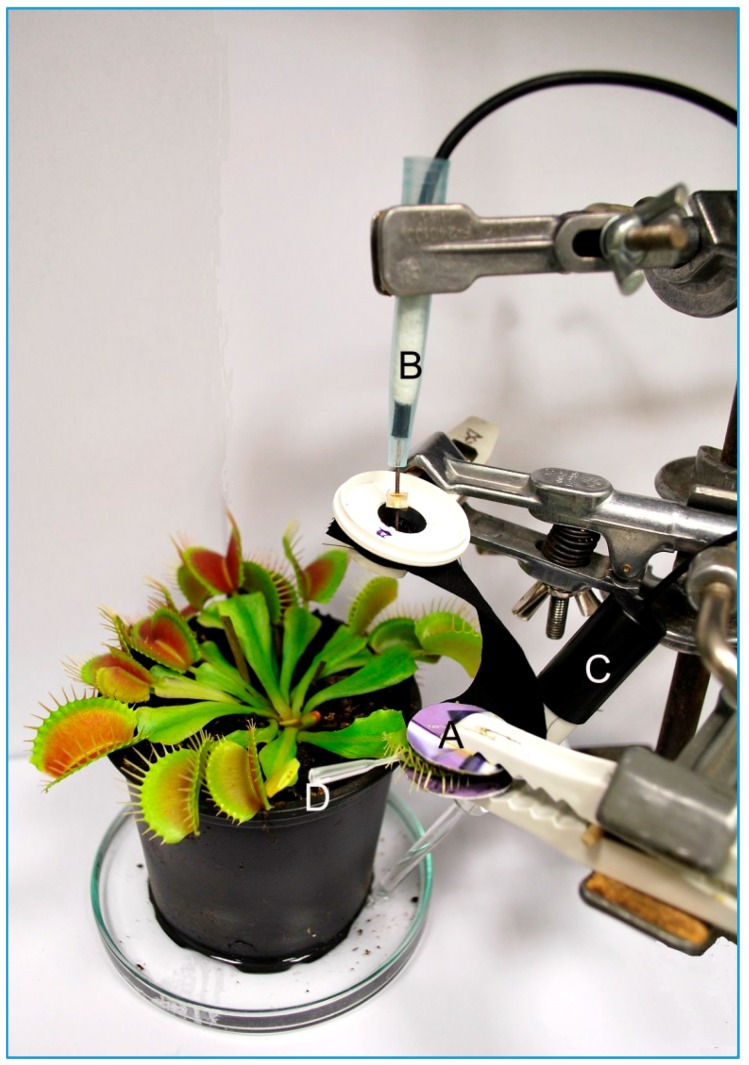
The layout of the electrodes used for recording of action potentials: clip electrode with cotton fabric coated with conducting polymer (**A**); non-polarizable Ag/AgCl surface electrode connected to the protruding strip of cotton fabrics coated with conducting polymer (**B**); and reference electrode (**C**); A plastic stick was used for stimulation of trigger hairs inside closed trap of Venus flytrap (**D**).

**Figure 8:**
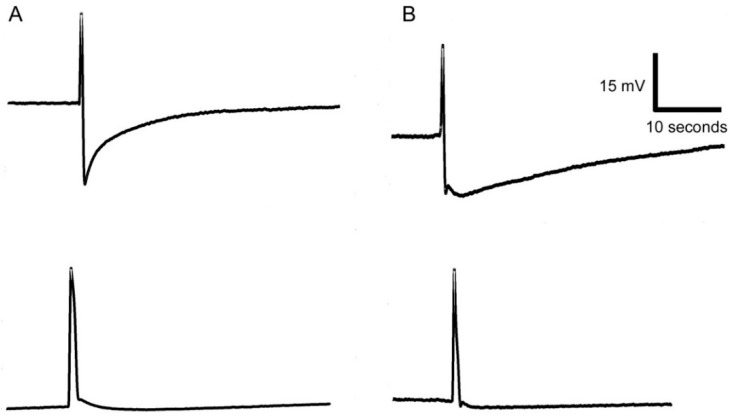
Action potentials recorded by electrode (**A**) and PANI-coated cotton fabric (**B**) in response to touching of trigger hair in Venus flytrap.

**Figure 9:**
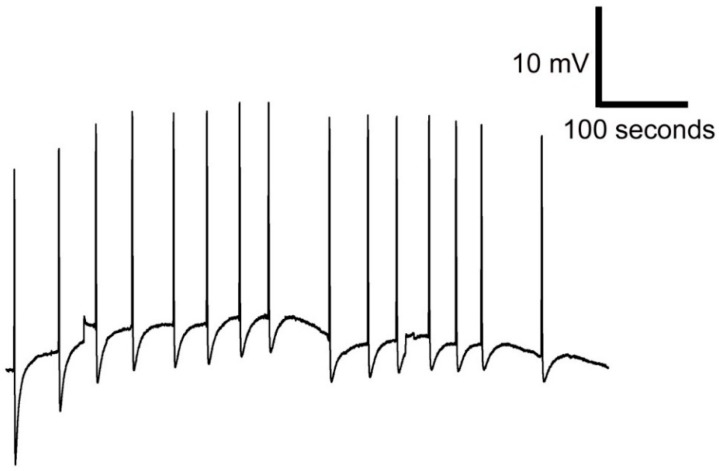
A series of action potentials of Venus flytrap in response to 15 touches of trigger hairs recorded by cotton fabrics coated with PPy+PANI.

The modified leaf of Venus flytrap called trap catches prey by rapid movement of its bilobed halves that shut when the trigger hairs protruding from the upper leaf epidermis are stimulated by touch. The stimulation of trigger hairs activates mechanosensitive ion channels and generates a receptor potential, which induces an action potential (AP). The struggling of the entrapped prey in the closed trap results in generation of further APs, which cease to occur when the prey stops moving [43]. We measured the AP in closed trap due to the stable geometric configuration of trap (in fact trap closure may results in loss of contact between trap tissue and electrode) (Figure 7). In Figure 8 you can see AP recorded by Ag/AgCl electrode (Figure 8A) and PANI-coated cotton fabric (Figure 8B). The most important thing is to make a good contact between the fabric and plant tissue to increase the signal-to-noise ratio and signal stability. The AP triggered by touch has a great variability, mainly in the degree of hyperpolarization and amplitude (Figure 8). The amplitude and hyperpolarization usually decreased with the consecutive number of APs triggered by touch (Figure 9). Nevertheless, you can see that the APs recorded using conductive polymers are comparable with that recorded by Ag/AgCl electrode connected to the plant surface by means of a conductive aqueous gel of the type commonly used in electrocardiography. The measurement using gel has often been considered as non-invasive but the usage of the osmotic conducting gel can cause serious tissue damage observable several hours/days after measurements. The present textile electrodes might avoid this drawback at expanse of the slightly increased noise. However, this can be overcome by data averaging if they were recorded with high sampling frequency.

In the preliminary testing, we have not observed any significant differences among fabrics coated with different conducting polymers: PANI, PPy, PANI+PPy, and PPy+PANI. All of them have been able to record the action potentials in similar quality of the plant to the repeated mechanical stimulation (data not shown) despite substantial differences in the sheet resistivity (Table 1). This is probably caused by the fact that the trap of Venus flytrap is a three-dimensional structure and contact area between the trap and coated fabric strongly varied during measurements from one another. The second possibility is that even the lowest conductivity is sufficiently high for recording of electrical signals in plants and is comparable than those measured using conductive gels.

The single coating of the textile with the conducting polymer is thus sufficient for the construction of the sensor. The higher conductivity afforded by the repeated coating of textile with conducting polymers, however, is likely to be of benefit for the improved collection and processing of the electrical response.

## 4. Conclusions

The cotton was uniformly coated with conducting polymers, polyaniline or polypyrrole, and by the combination of both polymers. The sheet resistivity decreased after the second coating with polypyrrole or polyaniline, respectively, due to the increased total amount of conducting polymer deposited on the fabrics, the best value being 210 Ω□^−1^ for the coating with polypyrrole followed by polyaniline. The conductivity of corresponding conducting polymers was of the order of units S·cm^−1^, both in parent polymers or their combined composites. No synergetic effect on the conductivity was observed. The repeated dry cleaning increased the sheet resistivity of fabrics by about one order of magnitude, but still maintained a good level of conduction, the best result being 1700 Ω^−1^, again for the twin polypyrrole/polyaniline coating. The fabrics were tested as electrodes in monitoring the electrical response of Venus flytrap stimulation. All cotton fabrics coated with both conducting polymers and their combinations have been able to collect and transfer the electrical response from Venus flytrap plant. No differences in this ability were observed among these coated fabrics. The usage of cotton fabric coated with polyaniline or polypyrrole conductive polymers may avoid the use of conducting gels at expense of slightly lower signal to noise ratio compared with Ag/AgCl electrodes. The relatively simple and cost-effective preparation process enables obtaining textiles with unique properties suitable for new specific end-use applications and attractive niche markets products development.

## Figures and Tables

**Table 1:** Sheet resistivity of cotton fabric coated with conducting polymers and the conductivity of such polymers.

Cotton	Sheet Resistivity, Ω□^−1^		Conductivity of Related Powders, S·cm^−1^
	as Prepared	after Dry Cleaning	
+PANI	6.3 × 10^4^	5.0 × 10^5^	2.2
+PANI+PPy	630	7.7 × 10^3^	7.2
+PPy	1.7 × 10^3^	5.0 × 10^3^	4.2
+PPy+PANI	210	1.7 × 10^3^	3.2
